# Diagnostic accuracy of the Scandinavian guidelines for minor and moderate head trauma in children: a prospective, pragmatic, validation study

**DOI:** 10.1016/j.lanepe.2025.101233

**Published:** 2025-02-13

**Authors:** Fredrik Wickbom, Rakel Bremell, Sarah Thornberg, Jorge Sotoca Fernandez, Beatrice Magnusson, Rasmus Silfver, Aqeel Chaudhry, Kristoffer Kjellröier, Hanna Farahnoosh Afsan, Marcus Bergman, Amel Jumppanen, Malin Johansson, Sascha Östberg, Christian Kamis, Mihai Ölund, Emma Jeppsson, Albert Modin, Anders Santoft, Lovisa Borg, Cathrine Gatzinsky, Maria Lönn, Olga Calcagnile, Ramona Astrand, Terje Sundstrøm, Niklas Marklund, Johan Undén, Scandinavian Neurotrauma Committee

**Affiliations:** a Department of Clinical Sciences, Malmö, Lund University Faculty of Medicine, Lund, Sweden; b Department of Operation and Intensive Care, Halland Hospital Halmstad, Region Halland, Halmstad, Sweden; c Department of Medicine, Halland Hospital Halmstad, Halmstad, Sweden; d Department of Paediatric Surgery, Queen Silvia Children's Hospital, Sahlgrenska University Hospital, Gothenburg, Sweden; e Paediatric Emergency Department, Department of Paediatrics, Skåne University Hospital, Malmö, Sweden; f Department of Diagnostics and Intervention, Anesthesiology and Intensive Care Medicine, Umeå University, Umeå, Sweden; g Department of Surgery, Halland Hospital Varberg, Region Halland, Halland, Sweden; h Regional Trauma Centre, Haukeland University Hospital, Haukeland, Bergen, Norway; i Department of Neurosurgery, Haukeland University Hospital, Haukeland, Bergen, Norway; j Karolinska University Hospital, Stockholm, Sweden; k Department of Emergency Medicine, Alingsås Hospital, Region Västra Götaland, Alingsås, Sweden; l Department of Surgery, Norra Älvsborgs Hospital, NU-sjukvården, Region Västra Götaland, Trollhättan, Sweden; m Department of Paediatric Medicine, Ryhov Hospital, Region Jönköpings län, Jönköping, Sweden; n Department of Surgery, Ljungby Hospital, Region Kronoberg, Ljungby, Sweden; o Department of Emergency Medicine, Ystad Hospital, Region Skåne, Ystad, Sweden; p Department of Surgery, Halland Hospital Halmstad, Region Halland, Halland, Sweden; q Department of Anaesthesia and Intensive Care, Mora Hospital, Region Dalarna, Mora, Sweden; r Department of Paediatrics, Skåne University Hospital, Lund, Sweden; s Institute of Clinical Sciences, Sahlgrenska Academy, Gothenburg University, Gothenburg, Sweden; t Department of Health and Care, School of Health and Welfare, Halmstad University, Halmstad, Sweden; u Psychiatry Halland, Region Halland, Halmstad, Sweden; v Department of Paediatric Medicine, Halland Hospital Halmstad, Halmstad, Sweden; w Department of Neurosurgery, Rigshospitalet, Copenhagen University Hospital, Copenhagen, Denmark; x Department of Clinical Medicine, University of Bergen, Bergen, Norway; y Department of Clinical Sciences, Lund University, Lund, Sweden; z Department of Neurosurgery, Skåne University Hospital Lund, Lund, Sweden

**Keywords:** Guidelines, Clinical decision rule, Traumatic brain injury, Children, Scandinavia, Validation, Diagnostic accuracy, Computed tomography

## Abstract

**Background:**

Current guidelines for initial management of traumatic brain injury (TBI) support decision making, but they are rarely validated. The Scandinavian guideline for management of children with TBI (SNC16) was developed to minimise the use of cranial computed tomography (cCT) without compromising safety, but the performance of the guideline in a real-world population is unknown. We aimed to determine the diagnostic accuracy for the SNC16 in a large, pragmatic cohort of children.

**Methods:**

In this prospective, observational, international cohort study in 16 Swedish and Norwegian emergency departments (EDs), children (aged <18 years) with blunt head trauma, presenting within 24 h of injury and a Glasgow Coma Scale of 9–15, were prospectively enrolled. The primary outcome measure was presence of a composite variable (clinically important intracranial injury (CIII) comprised of death, neurosurgery, admission to hospital ward ≥2 days due to head injury, or intubation ≥1 day due to pathological cCT findings), all within one week from trauma. Secondary outcome measures were neurosurgery and significant trauma related findings on cCT.

**Findings:**

A total of 3012 children were enrolled from April 2018 to May 2024. Nine patients fulfilled the primary variable CIII (0.30%; 9/3012), two patients required neurosurgery (0.07%; 2/3012), and 27 patients showed significant trauma related findings on cCT (0.90%; 27/3012). Point sensitivities to detect CIII, neurosurgery and significant cCT findings were 100% (CI 95% 70%–100% [9/9]; 34%–100% [2/2]; and 87%–100% [27/27]). Point specificity was 41.3%, 41.2%, and 41.6% (CI 95% 40%–43% [1241/3003]; 39%–43% [1241/3010]; and 40%–43% [1241/2985]). Negative predictive values were 100% for CIII, neurosurgery and significant cCT findings (CI 95% 99.7%–100.0% for all). Application of the SNC16 guidelines would have resulted in a mandatory cCT rate of 3.4% (101/3012) and immediate discharge from the ED for 41.2% (1241/3012) of children. No children with a discharge recommendation were positive for any primary or secondary outcomes.

**Interpretation:**

Validation of the SNC16 guideline showed adequate diagnostic performance in a real-world cohort, supporting formal implementation.

**Funding:**

Non-commercially (Swedish state) funded by Sӧdra Sjukvårdsregionen and Vetenskapliga Rådet, Hallands Hospital and 10.13039/501100009963Forskning och Utveckling, Halland.

**Research in context**

**Evidence before this study**

We searched MEDLINE, Scopus and CINAHL, without date or language restrictions, for studies relating to the validation of the Scandinavian guidelines for management of traumatic brain injury (TBI) in children (SNC16). The keyword search terms used were: (Scandinavian OR Nordic) AND (TBI OR traumatic head injury OR head trauma) AND children AND validation. After removal of duplicates, six papers were retrieved. Two of these were relevant, both post-hoc external validation studies in previously sampled cohorts from US and Australia/New Zealand, respectively. There were no prospective, validation studies reporting diagnostic test performance for the SNC16 guideline in the Scandinavian or real-world setting.

**Added value of this study**

In the largest ever prospective study of TBI in Scandinavia, the SNC16 guideline was shown to be diagnostically accurate in a real-world cohort of children. Mandatory cranial computed tomography (cCT) rates were low (3.4%). Over 40% of children could be discharged immediately from the emergency department, with none of these children experiencing any complications.

**Implications of all the available evidence**

These results allow for formal implementation and clinical use of the SNC16 guidelines for management of children with TBI, facilitating safe management, whilst minimising the use of cCT.

## Introduction

Each year, a large number of children are assessed in emergency departments (EDs) due to traumatic brain injury (TBI).^1^ Most of them are classified as having a mild TBI (mTBI), defined as a Glasgow Coma Scale (GCS) score of 13–15.^1^ A minority may suffer from life-threatening intracranial complications, such as intracranial haematomas, requiring urgent management. Assessment and triage of these children for further investigations, such as cranial computed tomography (cCT) and in-hospital observation, is a clinical challenge, often managed by the most inexperienced doctors.^2^ Investigation with cCT involves ionising radiation, with reports estimating a risk of at least 1 extra case of brain cancer and one case of leukaemia per 10,000 cCTs.^3^ In-hospital observation has been found to be equally efficient in detecting complications,^4^ although more expensive.^5^ Recent data indicate an overuse of cCT in paediatric head trauma, although implementation of clinical practice guidelines may reduce unnecessary diagnostic imaging.^6^^,^^7^

Substantial efforts aiming to support clinical decision making, through development and distribution of clinical practice guidelines and decision rules, have been made in the last 15 years. Among these are the Children's head injury algorithm for the prediction of important clinical events (CHALICE), derived from a United Kingdom cohort of 22,772 children,^8^ the Paediatric Emergency Care Applied Research Network (PECARN) guideline, based on 42,412 children from the United States^9^ and the CATCH (Canadian Assessment of Tomography for Childhood Head injury) guideline, from a cohort of 3866 children from Canada.^10^ More recently, the Australian and New Zealand Guideline for Mild to Moderate Head Injuries in Children (PREDICT) and the updated Head injury: assessment and early management guideline from National Institute for Health and Care Excellence (NICE23) were presented.^11^^,^^12^ All these guidelines differ in aim, structure, predictor variables and outcome measures, making comparisons difficult.

The management routines for paediatric head injuries in Scandinavia were explored in a cross-sectional survey in 2006.^13^ A lack of standardisation between hospitals and a need for management recommendations was identified. As a result, the Scandinavian guideline for management of minor and moderate head trauma in children (SNC16) was developed in an evidence- and consensus-based process within the Scandinavian Neurotrauma Committee group, primarily with the Scandinavian health care system in mind.^14^ The primary aim of this guideline is to identify patient-important outcomes in children with mild to moderate TBI (ED GCS of 9–15). Patients are risk stratified into five groups, from minimal to moderate risk TBI, based on risk factors from patient history and clinical examination. Recommended management, based on this stratification, may involve mandatory cCT, optional cCT or admission, extended observation or discharge. The SNC16 has been evaluated in other patient populations with good diagnostic performance.^15^^,^^16^ Recent reports indicate a high use of the SNC16 guideline among Swedish ED physicians and a high degree of passive dissemination.^2^^,^^17^ Before formal implementation, however, the guidelines should be validated in a representative, real-world cohort, as the majority of evidence used to construct the guidelines derive from international cohorts.^14^^,^^18^

The primary objective of this study was to determine the real-world diagnostic accuracy parameters for the SNC16 guideline when applied to a prospectively and pragmatically sampled cohort of children with TBI.

## Methods

### Study design

This is a prospective, observational, pragmatically sampled cohort of children with mild and moderate TBI from sixteen Swedish and Norwegian EDs. As a pragmatic study, we wanted to include the same cohort as would be applicable in real-life management, including all children seeking ED care for their head injury. Fifteen EDs (n = 15) were located in Sweden and one in Norway. Seven were university hospital EDs and three of these were dedicated children's hospitals. Five EDs were located in mid-size, regional hospitals and four in local hospitals. The recruitment period started in April 2018 and ended in May 2024, with final follow-up for diagnostic accuracy-specific endpoints completed in September 2024. Ethical approval was granted from the ethical review boards in Sweden and Norway, with informed oral consent obtained from a caregiver and the child when applicable (age >14 years). Detailed description of the study methods and statistical considerations are described in the previously published study protocol.^18^ The study was registered at ClinicalTrials.gov, NCT05964764. This report adheres to Standards for the Reporting of Diagnostic accuracy studies (STARD),^19^ see attached checklist (Supplementary File, Table S1).

### Participants

Patients 0–17 years old seeking ED care for TBI within 24 h of injury, with a GCS of 9–15 at initial assessment in the ED, were eligible for inclusion. Prehospital GCS was not recorded as the SNC16 (as with other guidelines) utilise GCS at ED assessment. Exclusion criteria were lack of informed consent, suspicion of non-accidental injury, penetrating head injury, lack of social security number in the participating country or inclusion in another study that could affect management/treatment in the ED. Patients fulfilling inclusion criteria were identified in the ED triage, or by the assessing physician. Informed consent was obtained from all patients following oral and written study information, in accordance with the ethical approval.

### Procedures

**Fig. 1:**
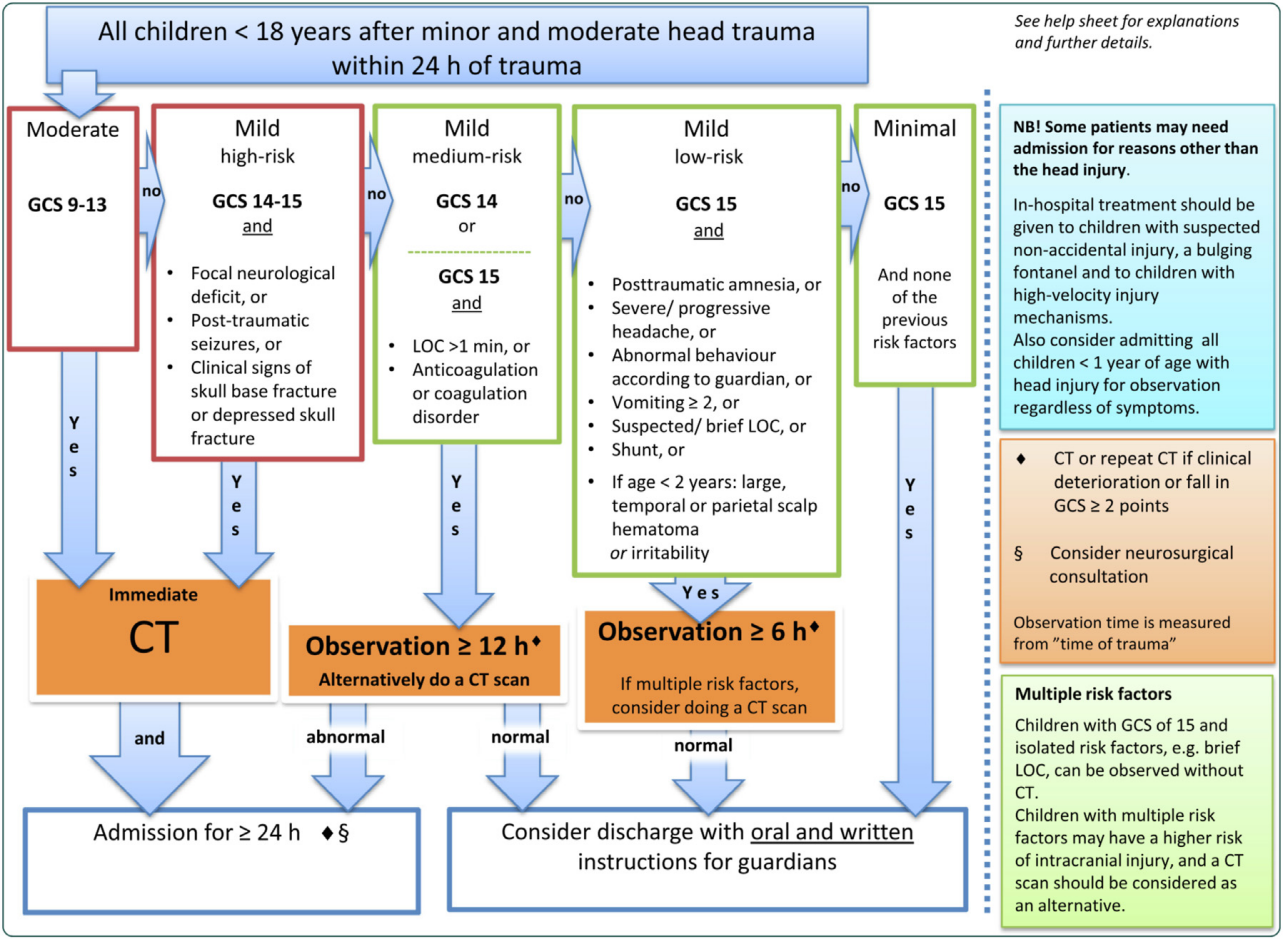
The flowchart accompanying the Scandinavian guidelines for management of minor and moderate head trauma in children (SNC16) is displayed. The SNC16 guideline aims to support clinicians in decision making when managing children with head trauma, specifically to identify patients in need for neurosurgical intervention or intracranial injury on cCT. The guideline is based on an evidence review and consensus process, developed specifically for use in Scandinavia. Children aged 0–17 years with blunt head trauma in the preceding 24 h and a GCS of 9–15 are classed in one of five risk groups based on clinical risk factors. The highest risk group are located to the left in the figure (*moderate risk TBI*), followed by the risk groups *mild-high risk TBI, mild-moderate risk TBI, mild-low risk TBI,* and *minimal risk TBI.* For respective risk groups there are concomitant recommendations on discharge, observation, cCT or a combination of these options. Adapted unchanged from Figure 6 in Astrand et al., 2016, Scandinavian guidelines for initial management of minor and moderate head trauma in children, BMC Medicine, distributed under CC BY 4.0 https://creativecommons.org/licenses/by/4.0/.^14^ Abbreviations: SNC16 guideline = Scandinavian guidelines for management of minor and moderate head trauma in children. cCT = cranial computed tomography. TBI = traumatic brain injury.

Participating units and their health care personnel were instructed to manage included patients in accordance with usual care. The index test evaluated is the Scandinavian guideline for minor and moderate head trauma in children, see Fig. 1.^14^ The primary endpoint was clinically important intracranial injury (CIII), within one week of injury, defined as a composite of death, neurosurgery, admission to hospital ward two days or more due to head injury, or intubation one day or more due to pathological traumatic CT findings. The use of the composite CIII harmonises with similar outcome measures applied in international research.^1^^,^^8^, ^9^, ^10^, ^11^, ^12^ Secondary outcomes were need for neurosurgery and significant cCT findings. Neurosurgery is defined as the need for any neurosurgical procedure or intervention, including neurointensive care with sedation, intubation and controlled ventilation for inoperable injuries, such as diffuse axonal injury, within 1 week of trauma. Significant cCT findings are defined as a trauma-related intracranial finding on CT scan (<1 week of trauma), such as intracranial haemorrhage or cerebral contusions, but excluding non-dislocated skull fractures. A complete review of all secondary outcomes can be found in the methodology publication.^18^ Data necessary for patient characterisation regarding risk factors and outcome were prospectively collected at several temporally separated points: 1) in the ED by the enrolling physician or nurse, 2) via medical records at ≥ 1 month from enrolment, and 3) via e-mail or text message to the caregiver(s) at 1, 3, and 4 months after injury.

As the SNC16 recommends different management paths depending on risk stratification, diagnostic test performance was calculated through three separate analyses, see Table 1. This allows for comparison of management paths, differentiating discharge versus all admission and cCT option, mandatory cCT versus no mandatory cCT and, finally, discharge and extended observation versus admission and cCT management.

**Table 1:** Test definitions for assessment of SNC16 diagnostic performance.




To evaluate test performance, a dichotomisation of the SNC16 guideline risk groups was made. Test performance is assessed with three different definitions of test positivity and test negativity (analysis 1, analysis 2, and analysis 3, above). For example, in analysis 1, patients classified to the minimal risk TBI group are assessed as test negative and patients in the other four risk groups are assessed as test positive.

Consecutive sampling was intended, but a proportion of eligible patients were anticipated to be missed. Screening logs were not deemed feasible in the study setting. To assess potential selection bias, a period of controlled sampling was performed where all patients could be accounted for. Details regarding test methods can be found in the full study protocol.^18^

### Statistical analysis

Relevant group statistics are presented descriptively. Sensitivity, specificity, positive and negative predictive values for SNC16's ability to predict the primary and secondary endpoints in the cohort are presented as point-estimates with 95% Wilson confidence intervals. The use of Wilson confidence intervals deviates from the planned use of Clopper-Pearson intervals, as it is more suitable for small sample sizes and furthermore adapted to use in multiple imputation datasets.^20^ Test results for analysis 1 are summarised in this manuscript and the complete results for analysis 1-3 are presented in the Supplementary File (Tables S8–S12). Rates for recommended mandatory cCT, optional cCT (observation or cCT), only observation and immediate discharge, according to the SNC16 guideline, are also presented, along with patients positive for a primary or secondary outcome missed by the guideline.

A best-case (where missing predictor variables are assumed negative) analysis, a complete case analysis and an analysis in a multiple imputation dataset are presented. The best-case analysis is deemed the most clinically relevant, as missing data on the index test most likely is negative in regard to the context of the data collection, as in a similar study by Babl et al.^21^ Hence, the best-case analysis is presented as the main analysis in this paper. If missing data are missing completely at random, the complete case analysis will report correct estimates with wider standard errors. If data are missing at random or missing not at random, the complete case analysis estimates may be biased, and multiple imputation is recommended.^22^ The multiple imputation assumes missing at random and may up-classify a patient's risk class. The possibility of introducing bias due to data being missing not at random is considered low when missingness is less than 20%,^23^ and thus unlikely to be an issue in the current study. Characteristics for missing data and specification of risk factors included in the imputation model are reported in the Supplementary File (Table S2). Regarding the number of datasets in the multiple imputation analysis, this number was greater than the proportion of patients with missing data, as outlined by Austin et al.^24^ An additional sensitivity analysis to address selection bias was conducted. Standard inclusion was compared to periods of controlled monitoring, where all eligible patients were documented with baseline data. In order to describe potential variability between centres, age groups, and arrival time to the ED, separate analyses regarding sensitivity and specificity are presented for these groups, including a forest plot (see Supplementary File Table S13 and Figures S14 and S15).

Entermedic (Entergate AB, Halmstad, Sweden) was used for data management and IBM SPSS Statistics (V.29) for statistical analyses. The multiple imputation Wilson confidence intervals were calculated according to the procedures described by Lott & Reiter.^20^

To obtain a sensitivity of 99% with a lower 95% confidence interval margin of 95%, with an estimated prevalence of the composite outcome in the population of 1.5%, including margins for loss to follow-up, the sample size was approximated to 5300 patients.^18^ An interim analysis at 1000 included patients indicated a significantly lower prevalence of the primary endpoint in the sample. The active recruitment period was predetermined to extend not more than four aggregated years, as results were requested to ensure safety of the already widely used SNC16 rule.^2^

Further details regarding statistical considerations can be found in the published protocol.^18^

### Role of the funding source

The funder of the study had no role in study design, data collection, data analysis, data interpretation, or writing of the report.

## Results

**Fig. 2:**
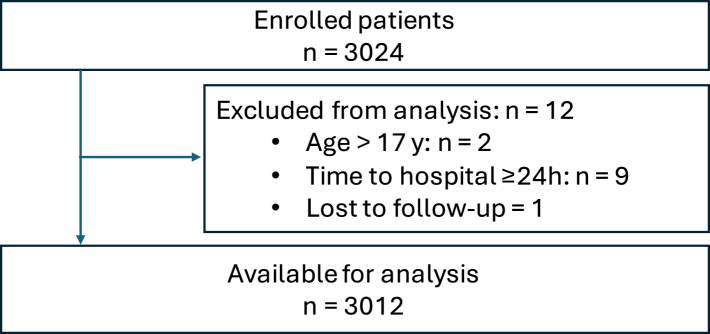
Flowchart describing enrolment and exclusion of non-eligible patients.

A total of 3024 patients were enrolled in the study, with the last patient included on the 26th May 2024. After exclusion of patients not fulfilling inclusion criteria (n = 11) and patients lost to follow-up (n = 1), 3012 patients remained for analysis (Fig. 2). The majority of patients were included in Sweden (2891/3012; 96%). The number of patients enrolled per year and per respective hospital are reported in the Supplementary Files; Tables S3 and S4. No patients were excluded due to suspected non-accidental injury and no competing studies were registered at any of the centres during the study period.

Detailed cohort characteristics are presented in Table 2. Mean age was 5.6 years (standard deviation 4.8) and almost one third (29.0%) of the patients were less than 2 years old. The ages of the children over the study period were uniform, see Supplementary File (Table S5). More boys than girls were enrolled (58.8% versus 41.2%) and falls were the most commonly reported trauma mechanism (68.0%). TBI severity, gender ratio and trauma mechanism, with respect to age, is presented in Supplementary File Table S6. Most patients had a GCS score of 15 in the ED (96.2%), with 12.6% reporting a period of loss of consciousness. Multiple SNC16 guideline risk factors were present in 21.5% of the patients. 1938 (64.3%) were discharged from the ED, 868 (28.8%) were kept in the ED for a period of observation, and 516 (17.1%) were admitted to an in-hospital ward. 219 children received a cCT (cCT rate of 7.3%), of which 27 showed significant CT findings (12.3% positive cCT rate, 0.9% of entire cohort). Six (n = 6) patients only had a linear skull fracture on cCT. All cCT findings are reported in the Supplementary File (Table S7). No patients died or were intubated for more than one day for their TBI. Nine patients (0.3%) fulfilled the composite endpoint CIII; all were admitted to a ward for more than 2 days due to head injury and 2 (0.07%) also had neurosurgery (evacuation of epidural haematoma and elevation of depressed skull fracture, respectively).

**Table 2:** Demographical, clinical and outcome cohort characteristics (n = 3012).

**Age and sex (number of patients with valid data, when missing data occurs)**	
Mean age	5.6 (SD 4.8)
Age <1 year	424 (14.1%)
<2 years	873 (29.0%)
≥2 years	2139 (71.0%)
Boys	1770 (58.8%)
Girls	1242 (41.2%)
**Trauma mechanism**^a^	
Fall	2048 (68.0%)
Sports	358 (11.9%)
In traffic	217 (7.2%)
Head hits stationary object	196 (6.5%)
Hit by moving object (low speed)	55 (1.8%)
Head hit by projectile or object in high speed	51 (1.7%)
Run into/collided with another person	49 (1.6%)
Assault	19 (0.6%)
Unknown/other mechanism	19 (0.6%)
**Clinical characteristics**	
Trauma alarm activated according to criteria for high velocity injury mechanisms	80 (2.7%)
GCS 9–13	24 (0.8%)
GCS 14	90 (3.0%)
GCS 15	2898 (96.2%)
Loss of consciousness (n = 3005)	380 (12.6%)
No LOC	2625 (87.2%)
<5 s	97 (3.2%)
5 s–1 min	172 (5.7%)
1–5 min	51 (1.7%)
>5 min	8 (0.3%)
Unknown	52 (1.7%)
Headache (n = 2996)	963 (32.0%)
Severe (n = 2996)	25 (0.8%)
Progressive (n = 2912)	81 (2.7%)
Vomiting (n = 2991)	821 (27.3%)
1 time	298 (9.9%)
2 times	196 (6.5%)
3 times	139 (4.6%)
4 or more times	172 (5.7%)
Abnormal behaviour according to guardian (n = 2892)	561 (18.6%)
Posttraumatic amnesia (n = 3001)	395 (13.1%)
Shunt	1 (<0.01%)
Scalp haematoma (n = 2998)	591 (19.6%)
Large (>3 cm) (n = 2996)	91 (3.0%)
Frontal (n = 2997)	343 (11.4%)
Parietal or temporal (n = 2997)	133 (4.5%)
Occipital (n = 2997)	114 (3.8%)
Clinical signs of skull base fracture (n = 3006)	9 (0.3%)
Depressed skull fracture	3 (0.1%)
Post-traumatic seizure (n = 2994)	25 (0.8%)
Focal neurological motor or sensory deficit (n = 2993)	25 (0.8%)
Abnormal pupils (n = 3000)	13 (0.4%)
Ataxia (n = 3004)	4 (0.1%)
Aphasia (n = 2999)	4 (0.1%)
Anticoagulation	1 (<0.1%)
Coagulation disorder	8 (0.3%)
Age <2 y and irritability	10 (0.3%)
Bulging fontanel (n = 2997)	0 (0%)
Multiple risk factors^b^	647 (21.5%)
**Outcomes**	
Cranial computed tomography	219 (7.3%)
Discharge from ED^c^	1938 (64.3%)
Prolonged observation in ED or ward^d^	868 (28.8%)
Admission to ward^c^	516 (17.1%)
Clinically important intracranial injury^e^	9 (0.3%)
Death	0 (0%)
Neurosurgery	2 (0.07%)
Admission to ward for 2 days or more due to head injury	9 (0.3%)
Intubation 1 day or more due to pathological traumatic CT findings	0 (0%)
cCT findings^f^	33 (1.1%)
Significant cCT findings^f^	27 (0.9%)

Age 0–17 years and <24 h since trauma. Continuous data are presented with mean and SD. Categorical variables are presented as number and percentages. Number of patients with missing data for these variables are presented in Supplementary File Table S2.

Abbreviations: GCS = Glasgow Coma Scale (paediatric GCS was reported for patients <5 years of age). LOC = loss of consciousness. cCT = cranial computed tomography. ED = emergency department.

a There were eight prespecified options for reporting of the trauma mechanism, including a free text option. Free text answers were then re-categorised by FW and JU into the prespecified categories or into two new categories. These were 1) hit by moving object (low speed) and 2) Run into/collided with another person. Where the free text reported trauma mechanism was deemed impossible to recategorize into any of the categories, the trauma mechanism remained classified as “other”.

b More than one of the risk factors presented in the SNC16 guideline flowchart (Figure 1).^14^

c As reported in medical records follow-up questionnaire.

d As reported by ED-physician or ED-nurse.

e Death, neurosurgery, admission to hospital ward 2 days or more due to head injury or intubation 1 day or more due to pathological traumatic CT findings.

f Significant CT findings are defined as a possibly trauma-related intracranial finding on CT scan, such as cranial fractures or acute intracranial haematoma, but not including undislocated skull fractures.

**Fig. 3:**
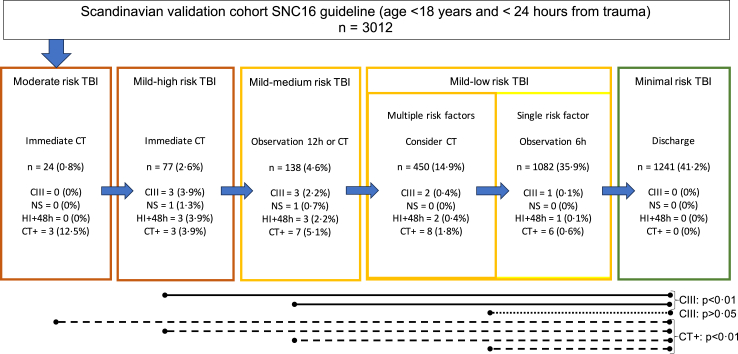
Distribution of children in the validation cohort in the SNC16 guideline risk groups. Number and percentage of patients positive for any outcome are displayed for respective group. Statistically significant differences for CIII and significant CT findings between groups are also shown (Chi-square test and Fischer's exact test when appropriate). Data shown for the best-case analysis. Patients in the mild-low risk group are sub-stratified on number of presented risk factors, single versus multiple, as this affects the SNC16 management recommendations. Abbreviations: CIII = clinically important intracranial injury. NS = neurosurgery. HI+48h = admission to hospital ward 2 days or more due to head injury. CT+ = significant cCT findings.

Cohort distribution and outcome when classified in SNC16 guideline categories are shown in Fig. 3. More than three quarters (77.1%; 2323/3012) of the children were classified either as minimal risk TBI or mild-low risk TBI with a single risk factor. Among these, one (n = 1) patient had a CIII (in-hospital care more than 2 days, due to a small subdural or epidural haematoma on cCT). This patient had age <1 year as the only SNC16 risk factor. Regarding the risk stratification of the SNC16, there were statistically significant differences in CIII and significant cCT findings when comparing patients with minimal risk TBI with higher risk groups (Fig. 3).

Point estimates and confidence intervals for prediction of the primary endpoint (CIII) and secondary endpoints neurosurgery and significant cCT findings in analysis 1 are presented in Table 3. Point sensitivities to detect CIII, neurosurgery and significant cCT findings in analysis 1 was 100%. Point specificity was 41.3%, 41.2%, and 41.6% for CIII, neurosurgery and significant cCT findings respectively, with similar point estimates and confidence intervals in the complete case analysis and in pooled data derived from multiple imputation. Negative predictive values were 100% for CIII, neurosurgery and significant cCT findings. Positive predictive values were less than 2%. Full results from the complete case, best case, and multiple imputation analysis are reported in the Supplementary File (Tables S8–S12). 110/3012 (3.65%) of patients had missing of at least in one SNC16 predictor variable necessary for index test classification.

**Table 3:** Diagnostic performance of the SNC16 guideline to predict CIII, neurosurgery and significant cCT + for patients with any SNC16 risk factor compared to patients with no risk factors (analysis 1; minimal risk TBI versus mild low, medium, high risk TBI, and moderate risk TBI).

Outcome	Diagnostic accuracy parameters	Best-case (n = 3012; missing index test variables assumed negative)	Complete case (n = 2902; 110 patients excluded due to at least one missing index test variable)	Pooled data from imputed datasets (n = 5)
Clinically important intracranial injury (n = 9)^a^	Sensitivity	100.0% (CI 95 70.1–100.0)	100.0% (CI 95 67.6–100.0)	100.0% (CI 95 70.1%–100.0%)
	Specificity	41.3% (CI 95 39.6–43.1)	41.4% (CI 95 39.6–43.2)	41.2% (CI 95 39.5%–43.0%)
	PPV	0.5% (CI 95 0.3–1.0)	0.5% (CI 95 0.2–0.9)	0.5% (CI 95 0.3%–1.0%)
	NPV	100.0% (CI 95 99.7–100.0)	100.0% (CI 95 99.7–100.0)	100.0% (CI 95 99.7%–100.0%)
Neurosurgery (n = 2)^a^	Sensitivity	100.0% (CI 95 34.2–100.0)	100.0% (CI 95 34.2–100.0)	100.0% (CI 95 34.2%–100.0%)
	Specificity	41.2% (CI 95 39.5–43.0)	41.3% (CI 95 39.5–43.1)	41.1% (CI 95 39.4%–42.9%)
	PPV	0.1% (CI 95 0.0–0.4)	0.1% (CI 95 0.0–0.4)	0.1% (CI 95 0.0%–0.4%)
	NPV	100.0% (CI 95 99.7–100.0)	100.0% (CI 95 99.7–100.0)	100.0% (CI 95 99.7%–100.0%)
Significant cCT+ (n = 27)^a^	Sensitivity	100.0% (CI 95 87.5–100.0)	100.0% (CI 95 86.7–100.0)	100.0% (CI 95 87.5–100.0)
	Specificity	41.6% (CI 95 39.8–43.4)	41.6% (CI 95 39.8–43.4)	41.5% (CI 95 39.7%–43.2%)
	PPV	1.5% (CI 95 1.0–2.2)	1.5% (CI 95 1.0–2.2)	1.5% (CI 95 1.0%–2.2%)
	NPV	100.0% (CI 95 99.7–100.0)	100.0% (CI 95 99.7–100.0)	100.0% (CI 95 99.7%–100.0%)

Abbreviations: PPV = positive predictive value. NPV = negative predictive value. CI 95 = 95% Wilson confidence interval.

a Best-case analysis.

**Fig. 4:**
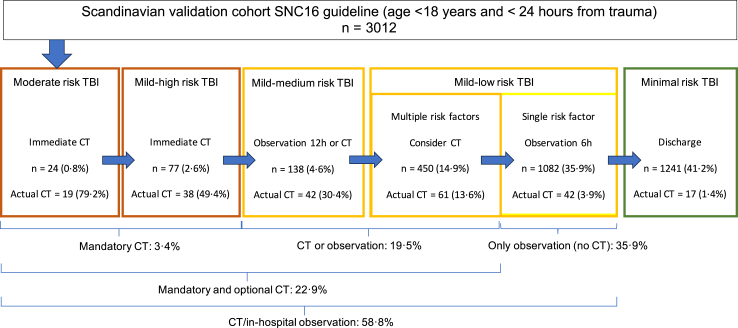
CT and observation rates when applying the SNC16 guideline. Actual CT rate in the cohort is also shown.

Application of the SNC16 guideline exhibited a mandatory cCT rate of 3.4%. If all children with cCT as an option in the guideline (mandatory and optional cCT) would have a cCT, it would result in a cCT rate of 22.9%. Observation without the need for cCT in the low-risk mild group, with a single risk factor, was recommended for 35.9% of patients (Fig. 4). In this group, one patient had a CIII and six patients had significant cCT findings, with none of these patients requiring neurosurgery or intubation. The negative predictive values for a patient classified as minimal (41.2% of all patients) or mild-low risk with a single risk factor (35.9% of all patients) were high (>99%) with lower 95% confidence intervals >99.4% for all three endpoints.

To assess the magnitude of a potential selection bias related to an unknown fraction of eligible but missed patients, periods of controlled inclusion were conducted in half of the participating centres (n = 8; three university hospital EDs, two mid-size hospital EDs, and three small hospital EDs) from January to June 2023. During 1–4 weeks per hospital (144 cumulated days), 152 eligible patients were enrolled, and 146 eligible patients were missed for inclusion. Differences regarding sex, age, SNC16 risk class and time of arrival to the ED (dichotomised to day 08–20 versus night 20–08) was explored in this group of 298 patients (missed versus included) and compared to the rest of the cohort. Results are presented in Table 4. Regarding the controlled inclusion period cohort, it was more common to miss inclusion of an eligible patient during night hours (n = 40; 27.4% versus n = 23; 15.1%, p = 0.01). Patients included during night hours were also more often classified as mild or moderate risk TBI (401/617; 65.0%), compared to patients included during daytime (1364/2395; 57.0%; p < 0.001). Regarding potential differences between the controlled inclusion period cohort and the standard inclusion cohort, patients in the latter had a higher SNC risk class (p < 0.001). No statistically significant differences were demonstrated regarding sex or age for missed versus included patients, nor for controlled versus the standard inclusion cohort. Performance variability between centres, age groups, and arrival time to the ED are presented in Supplementary File Table S13 and with forest plots in Supplementary File Figures S14 and S15.

**Table 4:** Sensitivity analyses regarding potential selection bias.

	Inclusion period		p-value	Entire cohort		p-value
	Missed (n = 146)	Included (n = 152)		Controlled inclusion period (n = 298)	Standard inclusion period^a^ (n = 2860)	
**Sex (male)**	90 (61.6%)	98 (64.5%)	0.613	188 (63.1%)	1672 (58.5%)	0.122
**Age**^b^	5.27; 4.76	5.47; 4.39	0.705	5.38; 4.57	5.57; 4.85	0.515
**Time of arrival to ED**						
*Arrived at night (20–08)*	40 (27.4%)	23 (15.1%)	**0.01**	63 (21.1%)	594 (20.8%)	0.880
*Arrived at day (08–20)*	106 (72.6%)	129 (84.9%)		235 (78.9%)	2266 (79.2%)	
**SNC16 risk class**^c^						
*Analysis 1 (minimal risk TBI)*	89 (62.2%)	83 (54.6%)	0.184	172 (58.3%)	1158 (40.5%)	**<0.001**

Statistical significance reported at p = 0.05 or less and marked bold in the table. Chi-square test, or Fischer's exact test when appropriate, was used for assessment categorical variables (sex, dichotomised time of arrival to ED, and dichotomised SNC16 risk class) and independent samples t-test for continuous, parametric variables (age).

a Patients included under the inclusion period (n = 152) are categorised to the controlled inclusion period group, with the standard inclusion period cohort comprising n = 3012–152 patients = 2860.

b Mean and standard deviation.

c SNC16 risk class are dichotomised as described for analysis 1 in Table 1, missing predictors presumed negative. Three patients (n = 3) in the missed group under the inclusion period (n = 146) lacked data on SNC-risk class.

## Discussion

We present the largest Scandinavian prospective TBI cohort to date, where the diagnostic accuracy of Scandinavian guidelines for initial management of minor and moderate head trauma in children was assessed in the intended real-world target population. The results strengthen previous findings from external validation studies,^15^^,^^16^ indicating that the SNC16 has high point sensitivities and negative predictive values for clinically relevant outcomes. These results support widespread clinical implementation and use of the SNC16 guidelines in Scandinavia.

The negative predictive value for patients in the minimal risk TBI group was 100% (95% confidence intervals >99%) for all endpoints; CIII, neurosurgery and significant cCT findings. The present SNC16 recommendation of discharge for the minimal risk TBI group is therefore feasible and clinicians can therefore be confident with this management. For the mild-low risk TBI group with a single risk factor, the recommendation of 6-h observation is also reasonable, as the risk of serious complication is very low. Recently, published guidelines of similar risk-groups have reduced the observation time to discharge to 4 h from trauma,^11^^,^^12^ although this strategy has not been validated. Since the guideline classifies each child into one of five risk groups, test characteristics can be assessed at various levels for test negativity and test positivity. The prevalence of CIII and significant CT findings were significantly higher for patients of higher risk, when compared to the minimal risk group, confirming the risk stratification of the SNC16 guideline. This stratification may aid clinicians to better consider complication risks in the different groups, better motivating the specific interventions.

The development of clinical decision rules for paediatric mTBI management^8^, ^9^, ^10^ have been driven by observations of excessive cCT use,^9^ along with cCT risks associated with ionising radiation,^3^ sedation,^25^ false-positives,^26^ and incidental findings.^27^ The baseline cCT referral rate was 7.3% in this study population and the mandatory cCT rate when applying the SNC16 guideline was 3.4%. In the APHIRST-cohort from Australia and New Zealand, the cCT baseline rate was 10% and in the original PECARN study (United States) the cCT rate was 35%.^9^^,^^21^ Proper implementation and adherence to a guideline has been shown to reduce the use of cCT in mTBI, especially if there is a high baseline cCT use.^28^^,^^29^ A cross-sectional report investigating use of neuroimaging in the United States reported a reduction of cCT use to 16.9% (95% confidence interval 16.7–17.1%).^7^ However, a recent systematic review and meta-analysis indicates an overutilisation cCT rate in PECARN low-risk TBI patients of 54% (95% confidence interval 20–89%) in some settings.^30^ Comparing the SNC16 with other decision rules in an external cohort, diagnostic performance was similar, but the mandatory cCT rate was lower for the SNC16.^16^ Specifically, the mandatory CT rate in this comparison was 5% for SNC16, 30% for CATCH, 22% for CHALICE. For PECARN, the mandatory CT rate is difficult to determine, as they (like SNC16) have both CT and observation options, but 47% were recommended CT or admission, compared to 42% for the same recommended management in SNC16.^16^^,^^21^ The trend and desire to reduce cCT utilisation may lead to a higher proportion of patients observed in EDs or admitted to hospital. Considering the potential risks of cCT, mandatory imaging should only be utilised for patients with higher risks of complications, for example, children with signs of skull base fractures, focal neurological deficits, post-traumatic seizures or GCS 9–13, or in cases where children clinically deteriorate or fail to improve.

The SNC16 guideline was recently evaluated in a systematic review of paediatric mild traumatic brain injury guidelines.^31^ Of 11 included guidelines, 6 were rated “high quality” on the overall score, including the SNC16 guideline. In two of the AGREE II domains, the SNC16 guideline displayed lower scores; the domains “applicability” and “editorial independence”. The applicability domain assesses if barriers and facilitators to application are described, if advice and/or tools for implementation are included, if potential resource implications are considered, and if monitoring/auditing criteria are presented. This has also been addressed in a recent publication investigating determinants for successful implementation of the SNC16 guideline in Swedish EDs.^17^ In future revisions of the guideline, implementation tools and descriptions of the underlying evidence can be improved. The flow chart structure/format seemed well-functioning as the guideline was perceived as practical and accessible to use, and hence worth to preserve. The editorial independence domain in AGREE II assesses whether views of the funding body have influenced the content, and if competing interests are reported and addressed. The Scandinavian Neurotrauma Committee includes TBI experts from Scandinavia and the group is completely independent from regulatory authorities or companies; hence this domain should not score poorly. This domain should therefore be thoroughly described in future revisions of the guideline.

There are several limitations when interpreting the results in this report. Although the cohort size is relatively large (comparatively larger than other cohorts considering the much lower populations of the Scandinavian countries), only 9 patients were positive for the primary endpoint CIII and only 27 patients were positive for significant cCT findings. The confidence intervals around the point estimates are therefore generally wide. Compared to the assumed incidence of 1.5% for the primary outcome in the sample size calculation, the incidence of CIII in this study is considerably lower (0.30%). This reasonably represents the difference in selection bias between studies. For example, in the PECARN cohort, patients with “trivial injury mechanism” were excluded.^9^ The present study reflects the real-world situation in Scandinavian EDs, where even trivial injuries are managed, where a clinical guideline should be expected to include all patients. Also, measures such as new regulations on helmet use, improvements in traffic environments and enhanced vehicle safety may have decreased the incidence of clinically relevant complications after paediatric TBI. If so, this may impact the design of future versions of a clinical practice guideline for mTBI management in Scandinavia. Considering the data from this study, measures to increase specificity would be the focus in a future guideline. This would lead to even fewer CT scans (and/or ward admissions), but these measures would need to be carefully weighed against the possibility of a reduction in sensitivity.

The cCT rate of 7.3% could result in verification bias, as some children may have asymptomatic intracranial pathology. However, the diagnosis of these would not change initial patient management. Also, a study including mandatory cCT in children would not be ethically viable. This aim of this study is to validate the SNC16 guidelines for emergency management of these children, which seeks to minimise cCT and admission without missing important complications. However, many children with seemingly mild TBI experience long-term symptoms which can have a profound impact on the child and the child's family.^32^ Although identification of functional long-term outcome is not part of the SNC16, this aspect would ideally have more focus in future updates.

Missing data implies a significant statistical obstacle and was unavoidable due to the large number of respondents in various contexts for each enrolled patient in this study. Efforts to map patterns of missing data, as well as various statistical approaches to depict the range of uncertainty, were used in this study. One could argue that multiple imputation should form the primary analysis. However, our approach best mimics the clinical reality. Additionally, missing data was uncommon and there were no major differences in the results for the complete case analysis, best-case analysis and the multiple imputation dataset analysis, which verifies the robustness of our results. Regarding variability, specificity was lower for children under 2 years of age. This is reasonably due to the SNC16 recommending admission for children under 1 year of age.

The results are mainly valid in a Scandinavian context although also relevant for other settings with similar patient populations,^21^ where the SNC16 guideline have also been externally validated.^16^ Future revisions should reflect the latest evidence within the area of paediatric mTBI management, also including often overlooked aspects of implementation science.

## Contributors

FW and JU conceived and planned the study. FW developed the digital CRF system. FW recruited and coordinated study centres in Sweden and Norway. ST, JSF, BM, RS, AC, KK, HF, MB, AJ, MJ, SÖ, AS, LB, CG, RB, and FW coordinated enrolment and medical records follow up at respective participating unit. CK, MÖ, EJ, and AM coordinated enrolment at participating centre. FW and RB compiled the data. FW conducted the statistical analysis with support from ML and RB. FW, ML, and JU interpreted the data. FW and JU wrote the initial draft, which was critically reviewed and approved by all authors. JU, NM, TS, OC, and RA provided overall supervision. TS, FW, AC, and JU wrote and applied for ethical permission in Sweden and Norway. All authors gave final approval to be published and agreed to be accountable for all aspects of the work.

## Data sharing statement

Pseudonymised datasets will be available on reasonable request from the corresponding author for at least 10 years from end of inclusion.

## Declaration of interests

All authors have completed the ICMJE uniform disclosure form at www.icmje.org/disclosure-of-interest/ and declare: No support from any organisation for the submitted work; no financial relationships with any organisations that might have an interest in the submitted work in the previous three years. JU, OC, RA, TS, and NM are all members of the SNC committee, a non-profit organisation independent from financial company support, who are responsible for the SNC16 guideline. JU and RA participated in the development of the SNC16 guideline. NM reports consulting fees from Teqcool Inc and honoraria from Balt Inc, with none of these having any connection with the present study, and is an unpaid board member of the European Neurotrauma Organisation and the European Association of Neurosurgical Societies. BM received registration (from the organisers) for the Nordic Neurotrauma Conference, 2023 in Lund.
