# Supplementary material for: Diagnostic accuracy of the Scandinavian guidelines for minor and moderate head trauma in children: a prospective, pragmatic, validation study
Source: Lancet Reg Health Eur. 2025 Feb 13;51:101233. doi: 10.1016/j.lanepe.2025.101233 (PMC11997442; doi:10.1016/j.lanepe.2025.101233)

## Supplementary online material for:

Diagnostic accuracy of the Scandinavian guidelines for minor and moderate head trauma in children: a prospective, pragmatic, validation study

December 15, 2024

## Contents list


| Page | Content | Heading |
|---|---|---|
| 3 | Table S1 | STARD checklist |
| 4 | Table S2 | Missing data and multiple imputation |
| 5 | Table S3 | Enrolled patients at respective hospital |
| 5 | Table S4 | Number of enrolled patients per year |
| 5 | Figure S5 | Distribution of age for enrolled cases per inclusion year |
| 6 | Table S6 | Additional details on cohort characteristics regarding age distribution, and data on severity of injury, gender and trauma mechanism in various ages |
| 7-9 | Table S7 | Trauma related cCT findings and characteristics for respective patient |
| 10 | Table S8 | Best case analysis: Test results for analysis 1, 2 and 3 |
| 11 | Table S9 | Complete case analysis: Test results for analysis 1, 2 and 3 |
| 12 | Table S10 | Multiple imputation dataset. Distribution of patients in the respective SNC16 risk group in the pooled data |
| 12 | Table S11 | Patient distribution for Analysis 1, Analysis 2 and Analysis 3 in the pooled dataset |
| 12 | Table S12 | Diagnostic accuracy for the SNC16 guideline when applied to the imputed dataset |
| 13 | Table S13 | Variability in sensitivity and specificity for the SNC16 guideline between centers, age groups and arrival time to ED |
| 14 | Figure S14 | Forest plot showing sensitivity for the SNC16 guideline between centers, age groups and arrival time to ED |
| 15 | Figure S15 | Forest plot showing specificity for the SNC16 guideline between centers, age groups and arrival time to ED |

**Supplementary Table S1. STARD checklist**

| Section & Topic | No | Item | Reported on page # |
|---|---|---|---|
| **TITLE OR ABSTRACT** | | **Diagnostic accuracy of the Scandinavian guidelines for minor and moderate head trauma in children: a prospective, pragmatic, validation study** | **1** |
| | **1** | Identification as a study of diagnostic accuracy using at least one measure of accuracy (such as sensitivity, specificity, predictive values, or AUC) | 1 |
| **ABSTRACT** | | | |
| | **2** | Structured summary of study design, methods, results, and conclusions (for specific guidance, see STARD for Abstracts) | 3 |
| **INTRODUCTION** | | | |
| | **3** | Scientific and clinical background, including the intended use and clinical role of the index test | 5 |
| | **4** | Study objectives and hypotheses | 5 |
| **METHODS** | | | |
| *Study design* | **5** | Whether data collection was planned before the index test and reference standard were performed (prospective study) or after (retrospective study) | 6 |
| *Participants* | **6** | Eligibility criteria | 6 |
| | **7** | On what basis potentially eligible participants were identified (such as symptoms, results from previous tests, inclusion in registry) | 6 |
| | **8** | Where and when potentially eligible participants were identified (setting, location and dates) | 6 |
| | **9** | Whether participants formed a consecutive, random or convenience series | 7 |
| *Test methods* | **10a** | Index test, in sufficient detail to allow replication | 6-7 |
| | **10b** | Reference standard, in sufficient detail to allow replication | 6-7 |
| | **11** | Rationale for choosing the reference standard (if alternatives exist) | NA |
| | **12a** | Definition of and rationale for test positivity cut-offs or result categories of the index test, distinguishing pre-specified from exploratory | 6-7 |
| | **12b** | Definition of and rationale for test positivity cut-offs or result categories of the reference standard, distinguishing pre-specified from exploratory | 6-7 |
| | **13a** | Whether clinical information and reference standard results were available to the performers/readers of the index test | 6-7 |
| | **13b** | Whether clinical information and index test results were available to the assessors of the reference standard | 6-7 |
| *Analysis* | **14** | Methods for estimating or comparing measures of diagnostic accuracy | 7 |
| | **15** | How indeterminate index test or reference standard results were handled | 7 |
| | **16** | How missing data on the index test and reference standard were handled | 7 |
| | **17** | Any analyses of variability in diagnostic accuracy, distinguishing pre-specified from exploratory | 7 |
| | **18** | Intended sample size and how it was determined | 7 |
| **RESULTS** | | | |
| *Participants* | **19** | Flow of participants, using a diagram | 8 |
| | **20** | Baseline demographic and clinical characteristics of participants | 8 |
| | **21a** | Distribution of severity of disease in those with the target condition | 8 |
| | **21b** | Distribution of alternative diagnoses in those without the target condition | NA |
| | **22** | Time interval and any clinical interventions between index test and reference standard | NA |
| *Test results* | **23** | Cross tabulation of the index test results (or their distribution) by the results of the reference standard | 8-9 |
| | **24** | Estimates of diagnostic accuracy and their precision (such as 95% confidence intervals) | 8-9 |
| | **25** | Any adverse events from performing the index test or the reference standard | NA |
| **DISCUSSION** | | | |
| | **26** | Study limitations, including sources of potential bias, statistical uncertainty, and generalisability | 9-11 |
| | **27** | Implications for practice, including the intended use and clinical role of the index test | 9-11 |
| **OTHER INFORMATION** | | | |
| | **28** | Registration number and name of registry | 6 |
| | **29** | Where the full study protocol can be accessed | 6 |
| | **30** | Sources of funding and other support; role of funders | 8 |

**Supplementary Table S2. Missing data and multiple imputation**

| Variable | Missing n | Imputed[e] (M=multiple imputation model; P=presumed negative, single imputation used) | In MI-model |
|---|---|---|---|
| Sex | 0 | No | Predictor |
| Loss of consciousness | 7 | M | Dependent |
| Age less than 1 year | 0 | No | Predictor |
| GCS[a] | 0 | No | Pedictor |
| Headache – intensity | 16 | P | No |
| Headache – course | 19 | M | Dependent |
| Abnormal behaviour | 30 | M | Dependent |
| Number of vomits | 21 | M | Dependent |
| Amnesia | 11 | M | Dependent |
| Previously healthy[b] | 1 | M | Dependent |
| Scalphaematoma - size | 16 | M | Dependent |
| Scalphaematoma - location | 15 | M | Dependent |
| Medications[c] | 1 | M | Dependent |
| Trauma alarm activated according to criteria for high velocity injury mechanisms | 0 | No | Predictor |
| Abnormal behavior according to guardian | 30 | M | Dependent |
| Posttraumatic amnesia (age > 1 years) | 11 | M | Dependent |
| Irritability and age < 2 years | 0 | No | No |
| Clinical signs of skull base fracture[h] | 6 | P | No |
| Clinical signs of depressed skull fracture | 0 | No | No |
| Post-traumatic seizure | 18 | P | No |
| Focalneurological deficit (non-merged question) | 19 | P | No |
| Abnormal pupils[g] | 12 | P | No |
| Ataxia (age> 1 year) | 8 | P | No |
| Afasia (age> 1 year) | 13 | P | No |
| Anticoagulation | 0 | No | No |
| Bleeding disorder | 0 | No | No |
| Bulging fontanel | 15 | P | No |
| | | | |
| **Outcomes** | | | |
| Neurosurgery[d] | 0 | No | Predictor |
| Intubation[f] | 0 | No | Predictor |
| Cranial computed tomography | 0 | No | Predictor |
| Death | 0 | No | No |

[a]Total GCS score was derived from the GCS subscores, or if a subscore was missing from RLS (if RLS was reported as RLS 1).
[b]If reported healthy, then presence of coagulation disorder, medications or shunt were deemed absent.
[c] If reported as having no medications, then use of anticoagulation is considered as absent and patient healthy.
[d] If missing data for neurosurgery but CT reported as “No” or “Normal”, neurosurgery is considered as absent. If discharged by nurse from ED with GCS15, neurosurgery is considered absent.
[e]Variables positive in <1% of the cohort were excluded from the imputation model (except for predictors intubation and neurosurgery) and single imputation used with presumed negative values due to high uncertainty in specification of the imputation model. Death was included in the first MI-models tested, but was excluded in the model development process as the predictor did not add any additional value (zero positive outcomes) to the model.
[f]If discharged from ED or cCT “No”, intubation was considered negative.
[g] If discharged by nurse from ED, with a GCS of 15 and no CT, pupils were considered normal.
[h] If discharged by nurse from ED, with a GCS of 15 and no CT, signs of skull base fracture was deemed as absent.

*Abbreviations: GCS = Glasgow Coma Score. RLS-85 = Reaction Level Scale -85. ED = Emergency department.*

## Supplementary Table S3. Enrolled patients at respective hospital

| | Hospital | Size | Included patients n (%) |
|---|---|---|---|
| **Sweden** | | | |
| 1 | Queen Silvia Children's Hospital, Sahlgrenska University Hospital, Gothenburg, Sweden | University ED | 371 (12·3%) |
| 2 | Astrid Lindgren´s Childrens hospital, Solna, Stockholm, Sweden | University ED | 298 (9·9%) |
| 3 | Skåne University Hospital, Lund, Sweden | University ED | 365 (12·1%) |
| 4 | Skåne University Hospital, Malmö, Sweden | University ED | 163 (5·4%) |
| 5 | Norrland University Hospital, Umeå, Sweden | University ED | 257 (8·5%) |
| 6 | Örebro University Hospital, Region Örebro, Örebro, Sweden | University ED | 9 (0·3%) |
| 7 | Halland Hospital Halmstad, Region Halland, Halland, Sweden | Regional ED | 583 (19·4%) |
| 8 | Halland Hospital Varberg, Region Halland, Halland, Sweden | Regional ED | 241 (8·0%) |
| 9 | Ryhov Hospital, Region Jönköpings län, Jönköping, Sweden | Regional ED | 125 (4·2%) |
| 10 | Norra Älvsborgs Hospital, NU-sjukvården, Region Västra Götaland, Trollhättan, Sweden | Regional ED | 137 (4·6%) |
| 11 | Mälarsjukhuset i Eskilstuna, Region Sörmland, Eskilstuna, Sweden | Regional ED | 20 (0·7%) |
| 12 | Alingsås Hospital, Region Västra Götaland, Alingsås, Sweden | Local ED | 158 (5·2%) |
| 13 | Mora Hospital, Region Dalarna, Mora, Sweden | Local ED | 55 (1·8%) |
| 14 | Ystad Hospital, Region Skåne, Ystad, Sweden | Local ED | 64 (2·1%) |
| 15 | Ljungby Hospital, Region Kronoberg, Ljungby, Sweden | Local ED | 45 (1·5%) |
| **Norway** | | | |
| 16 | Haukeland University Hospital, Haukeland, Bergen, Norway | University ED | 121 (4·0%) |

## Supplementary Table S4. Number of enrolled patients per year

| Year of inclusion | Included patients (n; %) | |
|---|---|---|
| 2018 | 43 | 1·4% |
| 2019 | 561 | 18·6% |
| 2020 | 575 | 19·1% |
| 2021 | 597 | 19·8% |
| 2022 | 497 | 16·5% |
| 2023 | 728 | 24·2% |
| 2024 | 11 | 0·4% |

## Supplementary Figure S5. Distribution of age for enrolled cases per inclusion year

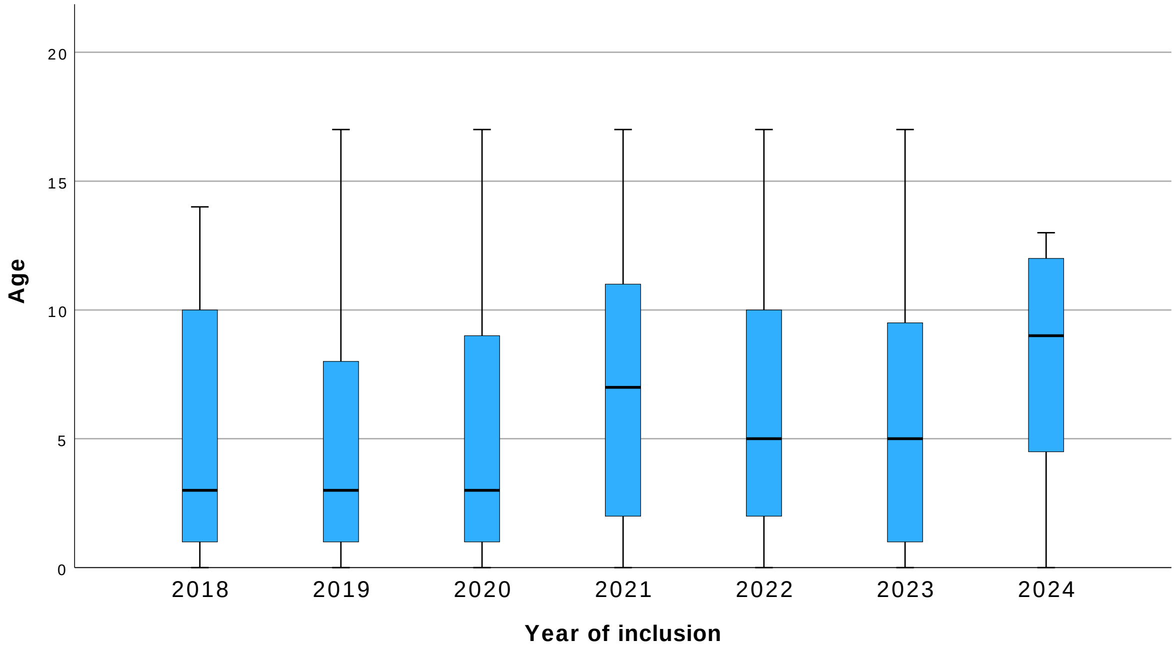

**Supplementary Table S6. Additional details on cohort characteristics regarding age distribution, data on severity of injury, gender and trauma mechanism for various ages**

| | 0-1 year | 2-4 year | 5-9 year | 10-13 year | 14-15 year | 16-17 year |
|---|---|---|---|---|---|---|
| **Patients in respective age category (n; %)** | 873 (29·0%) | 665 (22·1%) | 704 (23·4%) | 542 (18·0%) | 164 (5·4%) | 64 (2·1%) |
| **TBI severity (n; %)** | | | | | | |
| Minimal risk TBI | 248 (28·4%) | 367 (55·2%) | 345 (49·0%) | 209 (38·6%) | 57 (34·8%) | 15 (23·4%) |
| Mild-low risk TBI | 580 (66·4%) | 245 (36·8%) | 313 (44·5%) | 272 (50·2%) | 82 (50·0%) | 40 (62·5%) |
| Mild-medium risk TBI | 30 (3·4%) | 36 (5·4%) | 21 (3·0%) | 29 (5·4%) | 15 (9·1%) | 7 (10·9%) |
| Mild-high risk TBI | 13 (1·5%) | 9 (1·4%) | 20 (2·8%) | 25 (4·6%) | 8 (4·9%) | 2 (3·1%) |
| Moderate risk TBI | 2 (0·2%) | 8 (1·2%) | 5 (0·7%) | 7 (1·3%) | 2 (1·2%) | 0 (0·0%) |
| **Boy/girl ratio** | 1·3 : 1 | 1·4 : 1 | 1·7 : 1 | 1·4 : 1 | 1·4 : 1 | 0·8 : 1 |
| **Trauma mechanism** | | | | | | |
| Fall | 800 (91·6%) | 546 (82·1%) | 438 (62·2%) | 212 (39·1%) | 37 (22·6%) | 15 (23·4%) |
| Sports | 1 (0·1%) | 6 (0·9%) | 79 (11·2%) | 179 (33·0%) | 64 (39·0%) | 29 (45·3%) |
| In traffic | 5 (0·6%) | 21 (3·2%) | 62 (8·8%) | 77 (14·2%) | 41 (25·0%) | 11 (17·2%) |
| Head hits stationary object | 28 (3·2%) | 63 (9·5%) | 65 (9·2%) | 28 (5·2%) | 8 (4·9%) | 4 (6·3%) |
| Hit by moving object (low speed) | 16 (1·8%) | 13 (2·0%) | 19 (2·7%) | 5 (0·9%) | 1 (0·6%) | 1 (1·6%) |
| Head hit by projectile or object in high speed | 10 (1·1%) | 5 (0·8%) | 18 (2·6%) | 16 (3·0%) | 1 (0·6%) | 1 (1·6%) |
| Run into/collided with another person | 8 (0·9%) | 6 (0·9%) | 17 (2·4%) | 13 (2·4%) | 4 (2·4%) | 1 (1·6%) |
| Assault | 1 (0·1%) | 1 (0·2%) | 1 (0·1%) | 9 (1·7%) | 5 (3·0%) | 2 (3·1%) |
| Unknown/Other mechanism | 4 (0·5%) | 4 (0·6%) | 5 (0·7%) | 3 (0·6%) | 3 (1·8%) | 0 (0·0%) |

**Supplementary Table S7. Trauma related cCT findings and characteristics for respective patients**

| | Linear skull fracture | Depressed skull fracture (>1 bone width) | Skull base fracture | aSDH | EDH | tSAH | Contusions | Depressed fracture (< 1 bone width) | Other trauma related finding | Free text other finding | Age | Sex | Trauma mechanism | GCS | Intubation | Neurosurgery | Admitted >48h | SNC16 risk class |
|---|---|---|---|---|---|---|---|---|---|---|---|---|---|---|---|---|---|---|
| **Patients with trauma related "significant cCT-findings"** | | | | | | | | | | | | | | | | | | |
| 1 | No | No | No | No | No | No | No | **Yes** | **Yes** | Small haematoma, unclear if subdural or epidural. | 0 | Male | Fall | 15 | No | No | Yes | Mild-low risk with single risk factor |
| 2 | No | **Yes** | No | No | No | No | No | No | **Yes** | Depressed fracture 6 mm. Haematoma under fracture. | 2 | Female | Fall | 15 | Yes | Yes, elevation of depressed fracture | Yes | Mild-high risk |
| 3 | No | No | No | No | No | No | No | **Yes** | **Yes** | Extracranial haematoma. | 0 | Male | Fall | 15 | No | No | No | Mild-low risk with multiple risk factors |
| 4 | No | No | No | **Yes** | No | No | No | No | **Yes** | Radiologist: Suspected small amount of subdural blood along anterior falx. Neurosurgeon: Uncertain finding of blood along falx, not clinically significant. | 4 | Male | Fall | 15 | No | No | No | Mild-low risk with single risk factor |
| 5 | **Yes** | No | No | No | No | **Yes** | No | No | **Yes** | Discretely highly attenuating, cannot rule out small intracranial haematoma. Right tempoparietal extracranial haematoma. | 2 | Male | Fall | 14 | No | No | No | Mild-medium risk |
| 6 | **Yes** | No | No | No | No | No | No | No | **Yes** | 1-2 mm intracranial haematoma in connex to the fracture. | 0 | Male | Fall | 15 | No | No | No | Mild-low risk with multiple risk factors |
| 7 | No | No | **Yes** | No | No | No | No | No | No | | 1 | Male | Fall | 14 | No | No | No | Mild-medium risk |
| 8 | No | No | **Yes** | No | No | No | No | No | No | | 0 | Male | Fall | 15 | No | No | No | Mild-low risk with multiple risk factors |
| 9 | No | No | **Yes** | No | No | No | No | No | No | | 5 | Male | Fall | 15 | No | No | No | Mild-low risk with multiple risk factors |
| 10 | No | **Yes** | **Yes** | No | No | No | No | No | No | | 5 | Male | In traffic | 15 | No | No | No | Mild-low risk with single risk factor |
| 11 | No | No | No | No | No | No | **Yes** | No | No | | 11 | Female | In traffic | 15 | No | No | No | Mild-low risk with single risk factor |
| 12 | No | No | **Yes** | No | **Yes** | No | No | No | No | | 8 | Male | Fall | 15 | No | No | No | Mild-low risk with multiple risk factors |
| 13 | No | No | **Yes** | No | No | No | No | No | No | | 11 | Male | Fall | 14 | No | No | No | Mild-medium risk |

| | | | | | | | | | | | | | | | | | | |
|---|---|---|---|---|---|---|---|---|---|---|---|---|---|---|---|---|---|---|
| 14 | No | **Yes** | No | No | No | **Yes** | No | No | No | | 0 | Male | Fall | 14 | No | No | No | Mild-medium risk |
| 15 | No | No | **Yes** | No | **Yes** | No | No | No | No | | 11 | Male | Stationary object | 13 | No | No | No | Moderate risk |
| 16 | No | No | **Yes** | No | **Yes** | No | No | No | No | | 3 | Male | Fall | 13 | No | No | No | Moderate risk |
| 17 | No | No | **Yes** | No | No | No | No | No | No | | 12 | Female | Fall | 15 | No | No | No | Mild-high risk |
| 18 | No | No | No | **Yes** | No | No | No | No | No | | 13 | Male | Fall | 13 | No | No | No | Moderate risk |
| 19 | No | No | **Yes** | No | No | No | No | No | No | | 0 | Male | Fall | 15 | No | No | No | Mild-low risk with multiple risk factors |
| 20 | No | No | No | No | No | **Yes** | No | No | No | | 17 | Female | Fall | 15 | No | No | No | Mild-low risk with multiple risk factors |
| 21 | No | No | **Yes** | **Yes** | No | No | **Yes** | **Yes** | **Yes** | Small right sided brain contusion, subdural tentorial haematoma. Small parenchymal haematoma cannot be ruled out. Linear and depressed (less than one bone with) skull fractures. Extracranial haematoma. | 1 | Male | Fall | 15 | No | No | No | Mild-low risk with single risk factor |
| 22 | **Yes** | No | **Yes** | No | No | No | No | No | No | | 2 | Male | Hit by moving object | 14 | No | No | Yes | Mild-medium risk |
| 23 | No | No | No | No | **Yes** | No | No | No | No | | 10 | Male | Fall | 14 | Yes | Yes | Yes | Mild-medium risk |
| 24 | **Yes** | No | No | No | **Yes** | No | No | No | No | | 2 | Male | Fall | 15 | No | No | No | Mild-low risk with single risk factor |
| 25 | No | No | **Yes** | No | **Yes** | **Yes** | No | No | No | | 17 | Male | Fall | 15 | No | No | Yes | Mild-low risk with multiple risk factors |
| 26 | No | **Yes** | No | No | No | No | No | No | No | | 3 | Male | Fall | 15 | No | No | Yes | Mild-high risk |
| 27 | **Yes** | No | No | No | No | **Yes** | No | No | No | | 0 | Male | Fall | 14 | No | No | No | Mild-medium risk |
| **Patients with trauma related "cCT-findings"** | | | | | | | | | | | | | | | | | | |
| 28 | **Yes** | No | No | No | No | No | No | No | No | | 0 | Male | Fall | 15 | No | No | No | Mild-low risk with multiple risk factors |
| 29 | **Yes** | No | No | No | No | No | No | No | No | | 2 | Female | Fall | 14 | No | No | No | Mild-medium risk |
| 30 | **Yes** | No | No | No | No | No | No | No | No | | 0 | Male | Fall | 15 | No | No | No | Mild-low risk with multiple risk factors |
| 31 | **Yes** | No | No | No | No | No | No | No | No | | 2 | Male | Fall | 15 | No | No | No | Mild-low risk with single risk factor |
| 32 | **Yes** | No | No | No | No | No | No | No | No | | 0 | Male | Fall | 15 | No | No | No | Mild-low risk with multiple risk factors |
| 33 | **Yes** | No | No | No | No | No | No | No | No | | 6 | Female | Fall | 15 | No | No | No | Mild-low risk with single risk factor |

cCT findings are defined as a possibly trauma related intracranial finding on CT scan, such as cranial fractures or acute intracranial haemorrhage. Significant cCT findings are defined as a possibly trauma related intracranial finding on CT scan, such as cranial fractures or acute intracranial haemorrhage, but not including undislocated skull fractures.

**Supplementary Table S8. Best case analysis**

Analysis of 3012 patients, missing index variables were assumed negative.

| Outcome | Diagnostic accuracy parameters | Analysis 1 % (CI95) | Analysis 2 % (CI95) | Analysis 3 % (CI95) |
|---|---|---|---|---|
| **Clinically important intracranial injury** | Sensitivity | 100·0% (CI95 70·1 – 100·0) | 33·3% (CI95 12·1 – 64·6) | 66·7% (CI95 35·4 – 87·9) |
| | Specificity | 41·3% (CI95 39·6 - 43·1) | 96·7% (CI95 96·0 - 97·3) | 92·2% (CI95 91·2 – 93·1) |
| | PPV | 0·5% (CI95 0·3 - 1·0) | 3·0% (CI95 1·0 - 8·4) | 2·5% (CI95 1·2 – 5·4) |
| | NPV | 100·0% (CI95 99·7 – 100·0) | 99·8% (CI95 99·6 – 99·9) | 99·9% (CI95 99·7 – 100·0) |
| **Neurosurgery** | Sensitivity | 100·0% (CI95 34·2 – 100·0) | 50·0% (CI95 9·5 – 90·5) | 100·0% (CI95 34·2 – 100·0) |
| | Specificity | 41·2% (CI95 39·5 – 43·0) | 96·7% (CI95 96·0 – 97·3) | 92·1% (CI95 91·1 – 93·0) |
| | PPV | 0·1% (CI95 0·0 – 0·4) | 1·0% (CI95 0·2 – 5·4) | 0·8% (CI95 0·2 – 3·0) |
| | NPV | 100·0% (CI95 99·7 – 100·0) | 100·0% (CI95 99·8 – 100·0) | 100·0% (CI95 99·9 – 100·0) |
| **Significant cCT findings** | Sensitivity | 100·0% (CI95 87·5 – 100·0) | 22·2% (CI95 10·6 – 40·8) | 48·1% (CI95 30·7 – 66·0) |
| | Specificity | 41·6% (CI95 39·8 – 43·4) | 96·8% (CI95 96·1 – 97·4) | 92·4% (CI95 91·4 – 93.3) |
| | PPV | 1·5% (CI95 1·0 – 2·2) | 5·9% (CI95 2·8 – 12·4) | 5·4% (CI95 3.2 – 9·1) |
| | NPV | 100·0% (CI95 99·7 – 100·0) | 99·3% (CI95 98·9 - 99·5) | 99·5% (CI95 99·2 – 99·7) |

Analysis 1: Mild-low, mild-medium, mild-high and moderate risk TBI (positive test) versus Minimal risk TBI (negative test)
Analysis 2: Mild-high and moderate risk TBI (positive test) versus Minimal, mild-low and mild-medium risk TBI (negative test)
Analysis 3: Mild-medium, mild-high and moderate risk TBI (positive test) versus Minimal and mild-low risk TBI (negative test)
CI95 = Wilson 95% confidence intervals.

**Supplementary Table S9. Complete case analysis (n=2902 patients)**

110 patients were excluded due to at least one missing index test variable.

| Outcome | Diagnostic accuracy parameters | Analysis 1<br>% (CI95) | Analysis 2<br>% (CI95) | Analysis 3<br>% (CI95) |
|---|---|---|---|---|
| **Clinically important intracranial injury** | Sensitivity | 100·0%<br>(CI95 67·6 – 100.0) | 37·5%<br>(CI95 13·7 – 69·4) | 62·5%<br>(CI95 30·6 – 86·3) |
| | Specificity | 41·4%<br>(CI95 39·6 – 43·2) | 96·8%<br>(CI95 96·1 – 97·4) | 92·3%<br>(CI95 91·3 – 93·2) |
| | PPV | 0·5%<br>(CI95 0·2 – 0·9) | 3·1%<br>(CI95 1·1 – 8·8) | 2·2%<br>(CI95 0·9 – 5·0) |
| | NPV | 100·0%<br>(CI95 99·7 – 100·0) | 99·8%<br>(CI95 99·6 – 99·9) | 99·9%<br>(CI95 99·7 – 100·0) |
| **Neurosurgery** | Sensitivity | 100·0%<br>(CI95 34·2 – 100·0) | 50·0%<br>(CI95 9·5 – 90.5) | 100·0%<br>(CI95 34·2 – 100·0) |
| | Specificity | 41·3%<br>(CI95 39·5 – 43·1) | 96·7%<br>(CI95 96·0 – 97·3) | 92·2%<br>(CI95 91·2 – 93·1) |
| | PPV | 0·1%<br>(CI95 0·0 – 0·4) | 1·0%<br>(CI95 0·2 – 5·7) | 0·9%<br>(CI95 0·2 – 3·1) |
| | NPV | 100·0%<br>(CI95 99·7 – 100·0) | 100·0%<br>(CI95 99·8 – 100·0) | 100·0%<br>(CI95 99·9 – 100·0) |
| **Significant cCT findings** | Sensitivity | 100·0%<br>(CI95 86·7 – 100·0) | 24·0%<br>(CI95 11·5 – 43·4) | 44·0%<br>(CI95 26·7 – 62·9) |
| | Specificity | 41·6%<br>(CI95 39·8 – 43·4) | 96·9%<br>(CI95 96·2 – 97·4) | 92·5%<br>(CI95 91·4 – 93·4) |
| | PPV | 1·5%<br>(CI95 1·0 – 2·2) | 6·3%<br>(CI95 2·9 – 13·0) | 4·8%<br>(CI95 2·7 – 8·4) |
| | NPV | 100·0%<br>(CI95 99·7 – 100·0) | 99·3%<br>(CI95 98·9 – 99·6) | 99·5%<br>(CI95 99·1 – 99·7) |

Analysis 1: Mild-low, mild-medium, mild-high and moderate risk TBI (positive test) versus Minimal risk TBI (negative test)
Analysis 2: Mild-high and moderate risk TBI (positive test) versus Minimal, mild-low and mild-medium risk TBI (negative test)
Analysis 3: Mild-medium, mild-high and moderate risk TBI (positive test) versus Minimal and mild-low risk TBI (negative test)
CI95 = Wilson 95% confidence intervals.

**Supplementary Table S10. Multiple imputation dataset**

Distribution of patients in the respective SNC16 risk group in the pooled data (5 imputations).

| Moderate risk TBI n (%) | Mild-high risk TBI n (%) | Mild-medium risk TBI n (%) | Mild-low risk TBI n (%) | Minimal risk TBI n (%) |
|---|---|---|---|---|
| 24 (0.8%) | 77 (2.6%) | 138.4 (4.6%) | 1535.2 (51.0%) | 1237.4 (41.4%) |

**Supplementary Table S11. Patient distribution for Analysis 1, Analysis 2 and Analysis 3 in the pooled dataset**

| | Test positive | Test negative |
|---|---|---|
| Analysis 1 | 1774.6 (58.9%) | 1237.4 (41.1%) |
| Analysis 2 | 101 (3.4%) | 2911 (96.6%) |
| Analysis 3 | 239.4 (7.9%) | 2772.6 (92.1%) |

Analysis 1: Mild-low, mild-medium, mild-high and moderate risk TBI (positive test) versus Minimal risk TBI (negative test)
Analysis 2: Mild-high and moderate risk TBI (positive test) versus Minimal, mild-low and mild-medium risk TBI (negative test)
Analysis 3: Mild-medium, mild-high and moderate risk TBI (positive test) versus Minimal and mild-low risk TBI (negative test)

**Supplementary Table S12. Diagnostic accuracy for the SNC16 guideline when applied to the imputed dataset**

| Outcome | Diagnostic accuracy parameters | Analysis 1 % (CI95) | Analysis 2 % (CI95) | Analysis 3 % (CI95) |
|---|---|---|---|---|
| **Clinically important intracranial injury** | Sensitivity | 100·0% (70·1% - 100·0%) | 33·3% (12·1% - 64·6%) | 66·7% (35·4% - 87·9%) |
| | Specificity | 41·2% (39·5% - 43·0%) | 96·7% (96·0% - 97·3%) | 92·2% (91·2% - 93·1%) |
| | PPV | 0·5% (0·3% - 1·0%) | 0·3% (1·0% - 8·4%) | 2·5% (1·2% - 5·4%) |
| | NPV | 100·0% (99·7% - 100·0%) | 99·8% (99·6% - 99·9%) | 99·9% (99·7% - 100·0%) |
| **Neurosurgery** | Sensitivity | 100·0% (34·2% - 100·0%) | 50·0% (9·5% - 90·5%) | 100·0% (34·2% - 100·0%) |
| | Specificity | 41·1% (39·4% - 42·9%) | 96·7% (96·0% - 97·3%) | 92·1% (91·1% - 93·0%) |
| | PPV | 0·1% (0·0% - 0·4%) | 1·0% (0·2% - 5·4%) | 0·8% (0·2% - 3·0%) |
| | NPV | 100·0% (99·7% - 100·0%) | 100·0% (99·8% - 100·0%) | 100·0% (99·9% - 100·0%) |
| **Significant cCT findings** | Sensitivity | 100·0% (87·5 - 100·0) | 22·2% (10·6% - 40·8%) | 48·1% (30·7% - 66·0%) |
| | Specificity | 41·5% (39·7% - 43·2%) | 96·8% (96·1% - 97·4%) | 92·4% (91·4% - 93·3%) |
| | PPV | 1·5% (1·0% - 2·2%) | 5·9% (2·8% - 12·4%) | 5·4% (3·2% - 9·1%) |
| | NPV | 100·0% (99·7% - 100·0%) | 99·3% (98·9% - 99·5%) | 99·5% (99·2% - 99·7%) |

Analysis 1: Mild-low, mild-medium, mild-high and moderate risk TBI (positive test) versus Minimal risk TBI (negative test)
Analysis 2: Mild-high and moderate risk TBI (positive test) versus Minimal, mild-low and mild-medium risk TBI (negative test)
CI95 = Wilson 95% confidence intervals.

**Supplementary Table S13. Variability in sensitivity and specificity for the SNC16 guideline between centers, age groups and arrival time to ED**

| Analysis 1 versus clinically important intracranial injury (CIII) | | Sensitivity % (CI95) | Specificity % (CI95) | Number of patients in subgroup (of 3012) | % of cohort |
|---|---|---|---|---|---|
| **Age** | **0-1 years** | 100·0 (34·2 - 100·0) | 28·5 (25·6 - 31·6) | 873 | 29% |
| | **2-17 years** | 100·0 (64·6 - 100·0) | 46·6 (44·5 - 48·7) | 2139 | 71% |
| **Arrival time to ED** | **Day (06-18)** | 100·0 (64·6 - 100·0) | 42·8 (40·7 - 45·0) | 1996 | 66% |
| | **Night (18-06)** | 100·0 (34·2 - 100·0) | 38·4 (35·4 - 41·4) | 1016 | 34% |
| **Size ED** | **Local hospital** | NA* | 44·1 (38·8 - 49·6) | 322 | 11% |
| | **Regional hospital** | 100·0 (20·7 - 100·0) | 44·0 (41·1 - 46·9) | 1106 | 37% |
| | **University hospital** | 100·0 (67·6 - 100·0) | 38·9 (36·5 - 41·3) | 1584 | 53% |
| **Clustering** | **n<100** | NA* | 40·9 (34·2 - 48·0) | 193 | 6% |
| | **n=100-250** | 100·0 (43·9 - 100·0) | 44·6 (41·4 - 47·8) | 945 | 31% |
| | **n>250** | 100·0 (61·0 - 100·0) | 39·7 (37·5 - 42·0) | 1874 | 62% |

Clustering refers to the number of patients included at a unit during the study period. Data for sensitivity and specificity are also shown in supplementary figure S14 and S15, below.

*No patients positive for the outcome CIII.

*Abbreviations: ED = emergency department.*

**Supplementary Figure S14. Forest plot showing sensitivity for the SNC16 guideline between centers, age groups and arrival time to ED**

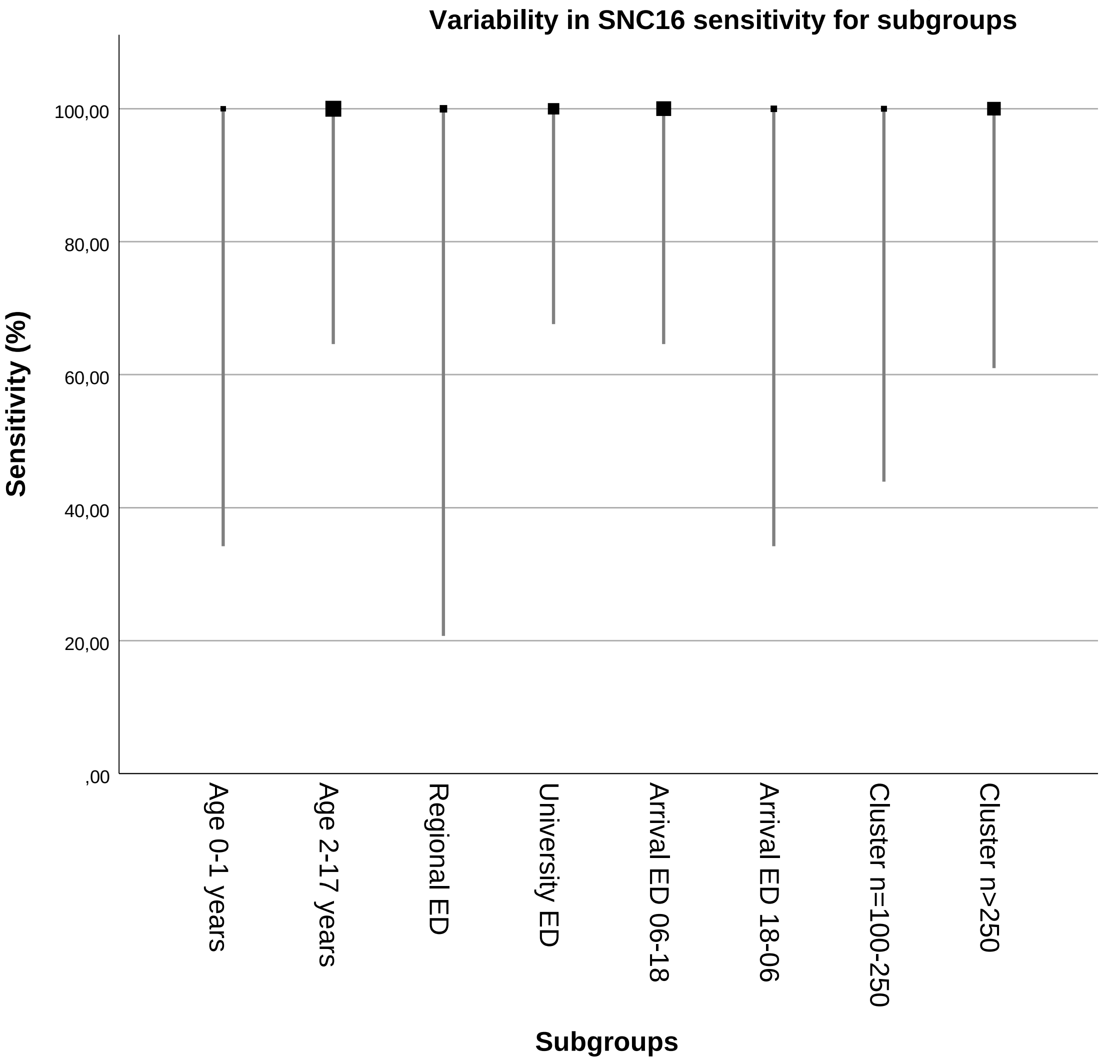

**Supplementary Figure S15. Forest plot showing specificity for the SNC16 guideline between centers, age groups and arrival time to ED**

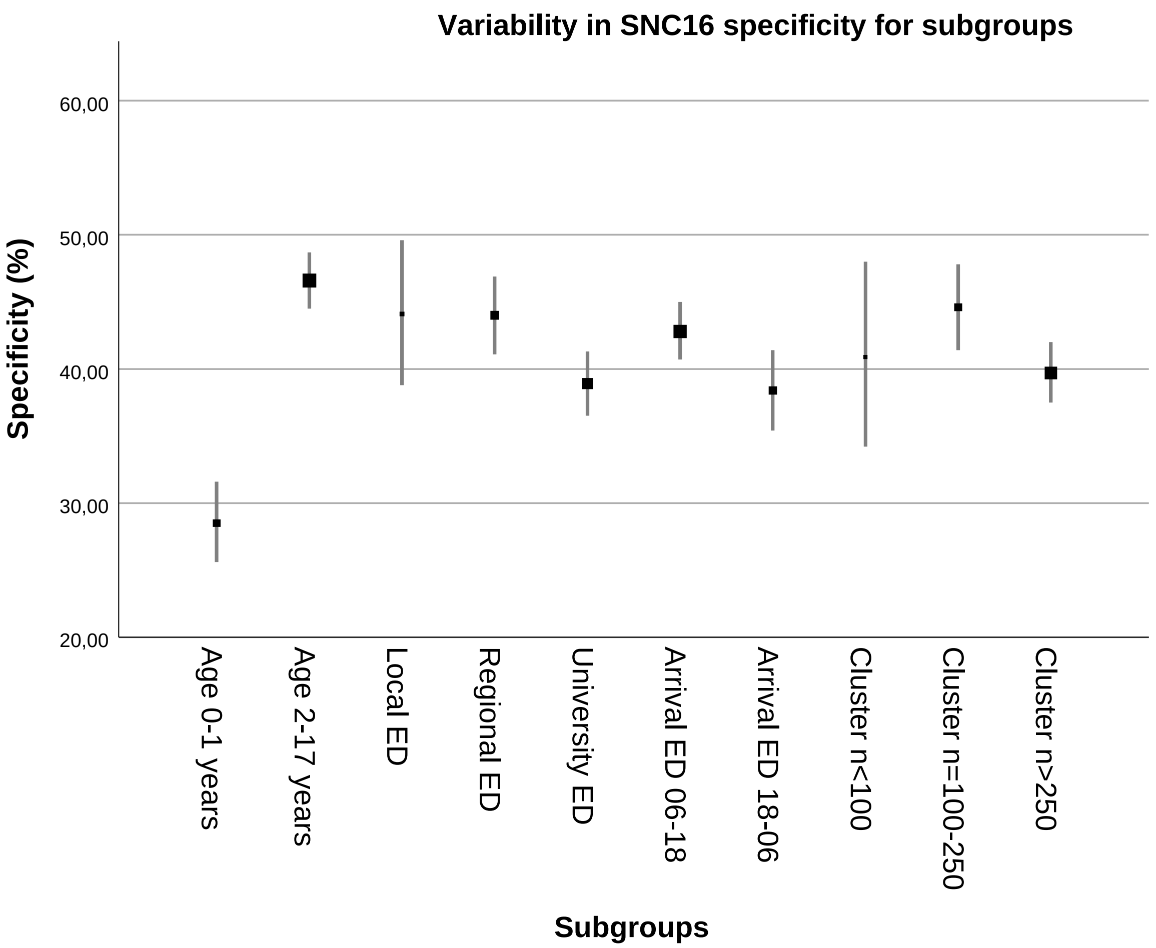

Supplement: Supplementary Materials [file mmc1.pdf]
